# A Comparison of Preoperative Predictive Scoring Systems for Postoperative Pancreatic Fistula after Pancreaticoduodenectomy Based on a Single-Center Analysis

**DOI:** 10.3390/jcm13113286

**Published:** 2024-06-03

**Authors:** Naomi Verdeyen, Filip Gryspeerdt, Luìs Abreu de Carvalho, Pieter Dries, Frederik Berrevoet

**Affiliations:** 1 Faculty of Medicine and Health Sciences, Ghent University, 9000 Ghent, Belgium; naomi.verdeyen@ugent.be; 2 Department of General and HPB Surgery and Liver Transplantation, Ghent University Hospital, 9000 Ghent, Belgium; filip.gryspeerdt@uzgent.be (F.G.); luis.abreudecarvalho@uzgent.be (L.A.d.C.); pieter.dries@uzgent.be (P.D.)

**Keywords:** risk assessment, pancreaticoduodenectomy, postoperative pancreatic fistula, preoperative predictive score

## Abstract

**Background**: Postoperative pancreatic fistula (POPF) after pancreaticoduodenectomy (PD) is associated with major postoperative morbidity and mortality. Several scoring systems have been described to stratify patients into risk groups according to the risk of POPF. The aim of this study was to compare scoring systems in patients who underwent a PD. **Methods**: A total of 196 patients undergoing PD from July 2019 to June 2022 were identified from a prospectively maintained database of the University Hospital Ghent. After performing a literature search, four validated, solely preoperative risk scores and the intraoperative Fistula Risk Score (FRS) were included in our analysis. Furthermore, we eliminated the variable blood loss (BL) from the FRS and created an additional score. Univariate and multivariate analyses were performed for all risk factors, followed by a ROC analysis for the six scoring systems. **Results**: All scores showed strong prognostic stratification for developing POPF (*p* < 0.001). FRS showed the best predictive accuracy in general (AUC 0.862). FRS without BL presented the best prognostic value of the scores that included solely preoperative variables (AUC 0.783). Soft pancreatic texture, male gender, and diameter of the Wirsung duct were independent prognostic factors on multivariate analysis. **Conclusions**: Although all predictive scoring systems stratify patients accurately by risk of POPF, preoperative risk stratification could improve clinical decision-making and implement preventive strategies for high-risk patients. Therefore, the preoperative use of the FRS without BL is a potential alternative.

## 1. Introduction

Recent improvements in surgical technique and perioperative management have reduced the perioperative mortality of pancreaticoduodenectomy (PD) to 2–7%. However, the complication burden remains high, ranging from 30 to 50% [1,2,3]. The leading cause of morbidity is postoperative pancreatic fistula (POPF), with a prevalence of 5–30% [4,5,6,7,8]. According to the International Study Group of Pancreatic Surgery (ISGPS), a clinically relevant POPF is defined as an abdominal drainage amylase level more than three times the upper limit of normal serum amylase activity on or after the third postoperative day and a clinically relevant change in the management of POPF [4]. POPF also contributes to secondary sequelae including delayed gastric emptying, post pancreatectomy hemorrhage, sepsis, multi-organ failure [7,9,10], and a subsequent mortality of up to 9% [10,11]. Therefore, POPF eventually results in a prolonged hospital stay and increased healthcare costs. Risk factors for POPF have been widely studied [8,12,13] and predictive scoring systems have been developed [2,7,10,11,14,15,16,17,18,19]. The Fistula Risk Score (FRS), which includes the intra- and postoperative variables of main pancreatic duct (MPD) diameter based on a surgeon’s measurement, gland texture based on a surgeon’s palpation, blood loss (BL) during surgery, and histopathology is the most extensively accepted and used risk score [20]. However, to improve surgical planning, risk management should consist of clinically relevant preoperative data to be able to implement preventive strategies to high-risk patients and reduce the incidence of POPF. In addition, parameters used in the risk models should be objectively and efficiently measurable. If not, even scores with excellent statistical predictability will not be useful in clinical practice.

The aim of this analysis was first to compare different prognostic POPF scoring systems and to determine their clinical feasibility in a large retrospectively analyzed cohort of patients after PD. Secondly, the aim was to compose a preoperative score to both identify high-risk patients and to be able to implement possible preventive strategies to reduce POPF.

## 2. Materials and Methods

### 2.1. Study Population and Data Collection

In this monocentric analysis, all consecutive 196 patients who underwent an open pylorus-preserving PD with a 4-layer interrupted duct-to-mucosa pancreaticojenunostomy for either chronic pancreatitis or premalignant (intraductal papillary mucinous neoplasm mucinous cystic neoplasm) and malignant lesions (neuroendocrine tumor, pancreatic ductal adenocarcinoma, ampullary carcinoma and cholangiocarcinoma) from July 2019 to June 2022 were included. No internal or external stenting was performed. Demographics, pre- and perioperative characteristics, and postoperative complications were recorded in a prospectively maintained database. POPFs were graded according to the ISGPF criteria [4], except lipase, which was measured instead of amylase at our institution [21,22,23]. The study was approved by the ethics committee of Ghent University Hospital (BC-08135) and performed according to the Declaration of Helsinki. First, an electronic search was performed on PubMed, Web of Science, and Google Scholar using the following terms as the search strategy: ‘preoperative’ AND [‘Whipple’ OR ‘pancreaticoduodenectomy’] AND [‘risk score’ OR ‘scoring system’ OR ‘prognostic score’ OR ‘risk assessment‘] AND ‘pancreatic fistula’. The aim was to identify studies suggesting a preoperative score to predict the probability of POPF after PD. To identify any other studies not yet included in the electronic search, the reference list of reviewed articles was also consulted. All studies that focused on preoperative scoring systems and that have been validated and used to predict postoperative pancreatic fistula (POPF) in human patients following pancreaticoduodenectomy (PD) were included. Eligible studies should have been conducted after 2013 and needed to report the area under the receiver operating characteristics curve (AUC). Studies that included both PD and (distal) pancreatectomy were excluded from our literature analysis, as were studies that included variables not present in our prospectively maintained database. Finally, the FRS and 4 validated preoperative scoring systems [7,18,19,20,24] were included in our analysis. An extra score was added by removing the intra-operative variable BL from the original FRS to determine if there was a significant impact on the predictive value of the FRS (FRS without BL).

### 2.2. Statistical Analysis

Statistical analysis was performed using SPSS version 28.0. Continuous data are presented as medians and ranges. Categorical variables are presented as frequencies with percentages. First, univariate and multivariate logistic regression was performed to assess preoperative predictors that are associated with POPF according to the risk scores. For multivariate analysis, all significant univariate variables were considered with significance level for inclusion at 0.05. To evaluate the efficacy of the different scoring systems, we merged patients that showed intermediate and high risk for developing POPF and eventually developed grade B–C POPF together to establish a test-positive group and patients with negligible risk and low risk and no development of POPF to form a test-negative group. The performance of all six scores in general and subgroups according to histopathology were evaluated by analyzing the AUC, positive predictive value (PPV), negative predictive value (NPV), sensitivity, and specificity.

## 3. Results

### 3.1. Literature Search

An overview of the studies is presented in Table 1. Perri et al. [7] proposed a regression risk-tree model which allocated patients into three risk groups based on body mass index (BMI) and MPD diameter. Roberts et al. [24] constructed a score based on the same variables as Perri et al. However, different stratification of the risk groups was present. Callery et al. [20] designed the FRS with a score from 0 to 10. The a-FRS by Mungroop et al. [18] excluded the variables ‘BL’ and ‘histopathology’, added BMI, and used radiological MPD diameters instead of the intraoperative measurement. Mungroop et al. [19] optimized this score by adding ‘gender’ to the a-FRS for patients undergoing minimally invasive PD and created the ua-FRS.

### 3.2. Cohort Characteristics

A total of 196 patients underwent open pylorus-preserving PD at our institution. The clinical characteristics of the cohort are reported in Table 2. The median age of all patients was 69 years. In total, 97 patients were male (49%), and 99 were female (51%). The median BMI was 24.6. Forty-eight percent of all patients were in ASA 3. Hard gland texture on MRI findings was observed in 64% of the patients, the median blood loss during surgery was 350 mL, and the median radiological MPD diameter was 4 mm. Most patients with an abdominal drainage lipase level more than three times the upper limit of normal serum lipase activity on or after the third postoperative day were given somatostatin analogs, and drains were left in place. These patients were considered as having a grade B fistula, because it is defined as a clinically relevant change in POPF management. Most patients had malignant lesions (79%). The minority of patients had premalignant lesions (13%), and only 8% had chronic pancreatitis. The overall 30-day mortality was 2.0%.

### 3.3. Validation and Comparison of Prognostic Scoring Systems

A two-stage method was performed for the external validation of the selected risk scores by applying uni- and multivariable analyses of the variables according to the cut-off levels of individual scores, followed by ROC analysis for an estimation of the predictive ability.

Upon univariable analysis (Table 3), a negative strength of association with risk of grade B-C fistula was seen for an increase in MPD diameter (OR 0.63, CI 0.50–0.79). A positive strength was identified in patients with a soft pancreatic texture (OR 6.86, CI 3.31–14.23), male gender (OR 2.06, CI 1.04–4.06), and histology other than pancreatic adenocarcinoma or pancreatitis (OR 3.53, CI 1.77–7.07). Patients with MPD diameter ≤ 1 mm had very high odds (OR 25.33, CI 2.00–321.31) of developing POPF. A lower degree of association was estimated for BMI ≥ 25 (OR 1.52, CI 0.78–2.96), ASA 2 (0.87, CI 0.21–3.63), ASA 3 (OR 0.55, CI 0.13–2.35), and blood loss > 1000 mL (OR 1.52, CI 0.37–6.19) without statistical significance (*p* = 0.217, *p* = 0.849, *p* = 0.422, *p* = 0.561, respectively).

On multivariate analysis, soft pancreatic texture (OR reaching 5.00 for Callery et al. [20]), MPD diameter (OR reaching 6.83 for Perri et al. [7]), and male gender (OR 2.35, CI 1.09–5.07) were positively associated with the occurrence of POPF.

**Figure 1:**
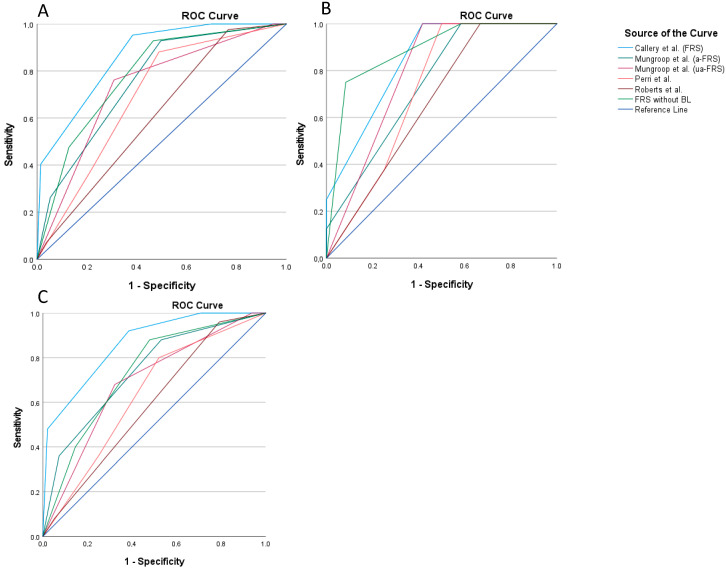
ROC analysis of the different scoring systems according to subgroups. (**A**) ROC for all patients; (**B**) ROC for premalignant lesions; (**C**) ROC for malignant lesions. a-FRS, adapted fistula risk score; BL, blood loss; FRS, fistula risk score; ua-FRS, updated alternative fistula risk score [7,18,19,20,24].

Table 4 shows an overview of the six scoring systems according to the different stratifications. A sufficient prognostic value was seen when the six scores were applied to our cohort (*p* < 0.001). We assessed the predictive value of the different scoring systems in patients with malignant and premalignant lesions separately (Figure 1). The original FRS showed the best performance for malignant lesions (AUC 0.862). However, the FRS without BL tended to be the best prognostic tool for premalignant lesions (AUC 0.885).

## 4. Discussion

This study first conducted a comprehensive overview of current available risk scores for POPF in the literature including the most used FRS. We validated these scores in a large cohort of PD patients and observed that all six models effectively categorized the patients into different risk levels (*p* < 0.001). Although the original FRS had the highest sensitivity and specificity compared to the other scoring systems, with an AUC of 0.862 (95% CI 0.89–0.97), a PPV of 43.6, and an NPV of 97.6, several variables of the FRS are obtained either during or after surgery, with the most influential predictor being the MPD.

In order to effectively predict POPF, it is necessary to incorporate only preoperative characteristics that are accessible and can be replicated. This will enable both the pre- and intra-operative strategy aimed at reducing the morbidity and mortality associated with POPF to be adapted. Multiple studies have already demonstrated a significant negative correlation between the width of the MPD and the incidence of POPF, as smaller duct conduits are more susceptible to micro-injury and leakage [8,12,13,25]. In addition, our findings demonstrate that the width of the MPD may be easily determined before surgery without reducing the predicted accuracy of the FRS. Furthermore, there is evidence indicating that the accuracy of predicting a grade B-C fistula using preoperative CT is comparable to using an intraoperative measurement of MPD [2,10,24]. This suggests that this test can be simply and accurately conducted in a clinical setting.

Moreover, soft pancreas parenchyma has previously been shown to have a strong association with the occurrence of pancreatic fistula [14,26]. It leads to increased enzyme secretion, whereas the intraoperative vulnerability makes the anastomosis difficult to perform [20,27]. However, using the FRS, the assessment of pancreatic texture is performed intraoperatively by the operating surgeon. There are already several alternatives to conquer this problem. First, BMI has been added as a new variable for the calculation of the a-FRS, the ua-FRS, as well as by Perri et al. and Roberts et al., because it is described as a positive predictor of soft pancreatic texture and steatosis and a negative predictor of fibrosis and vessel density [25,28,29,30]. Our study did not find a significant correlation with POPF, resulting in lower AUC values for scoring systems that include BMI. Therefore, more preoperative characteristics are required to forecast the pancreatic tissue. Previous reports have documented the quantification of pancreatic consistency using CT and MRI imaging techniques. Wong et al. established a specific criterion for a fatty pancreas, defining it as having a minimum of 10.4% fatty infiltration. This determination was made utilizing MRI with a 3D approach [31]. Lin et al. confirmed the accuracy of the a-FRS by utilizing CT patterns to assess gland texture [32]. In our investigation, all scoring systems were validated using MRI data to evaluate the texture of the pancreatic parenchyma. Integrating radiographic assessment as a standard method for evaluating the texture of the pancreas could get us closer to effectively managing the risk of POPF before surgery.

To our knowledge, this study is the first study to assess the prediction accuracy of scoring systems for various histological types of disease. It is crucial to potentially verify scoring methods that are customized for various histology kinds. Nevertheless, the evaluation of pathology with high precision is typically only accessible after the surgical procedure, rendering it incompatible as a predictive tool prior to surgery. When a biopsy is recommended, as was the case for 59% of our study participants, it can assist in distinguishing the histological characteristics before surgery, thus providing further benefit to the subgroup analysis. Furthermore, some investigations have found specific CT and MRI characteristics that can assist radiologists in making a definitive histological diagnosis with confidence [33,34].

Estimated BL during surgery is a well-known, but often inaccurate variable [35,36,37,38]. External validation as well as our analysis did not find it to be associated with POPF (*p* = 0.561), and the exclusion of this parameter did not change the predictive performance of the FRS in several studies [39,40,41]. Although the original FRS showed the best AUC, it involves several intra- and postoperative factors. In contrast, the FRS without the incorporation of BL could be easily determined preoperatively by means of radiological findings and biopsy, meaning this score could be more practical in the preoperative risk stratification of POPF. The ideal preoperative score to predict POPF in our study population would include the variables of MPD diameter, gender, and pancreatic texture because they were found to be significant predictive factors for POPF in the multivariate analysis.

Regarding the ability to preoperatively stratify the risk of developing POPF, preventive strategies can be implemented. Several studies have described the efficacy of drain placement in the prevention of autodigestion, which can lead to POPF. The PANDRA trial, an RCT including 395 patients, concluded that the frequency of POPF was lower in the patients without therapeutic lavage, with no significant difference in the general complication rate [42]. Another RCT by Fisher et al. did not complete the follow-up time because higher mortality was observed in the group without prophylactic drainage [43]. A meta-analysis reported similar results despite a higher occurrence of major morbidity [44]. Although the best timing for drain removal remains unclear, a subsequent prospective multicenter trial showed how early drain removal and selective drain placement is associated with better outcomes, especially in low-risk patients, as drains are a risk for retrograde intra-abdominal infection [45]. On the other hand, using intra-abdominal drainage in high-risk patients for grade B-C fistulas to reduce bacterial flora has been shown to reduce the incidence of intra-abdominal infections and could decrease the risk of POPF. These results show how crucial it is to properly stratify patients preoperatively for the risk for significant fistulas. The modern risk-based management of drain placement after PD is described by Marchegiani et al. [46]. In low-risk patients, drains are used at the discretion of the surgeon and can be removed. A drain fluid amylase level on postoperative day 1 below 5000 U/L is an indication for drain removal on postoperative day 3. If the drain amylase level exceeds 5000 U/L on postoperative day 1, if patients are in a high-risk group, or if an externalized transanastomotic stent is present, the amylase is tested after five postoperative days. If amylase exceeds three times the upper limit of normal, POPF is present.

Somatostatin analogs are well-known inhibitors of the exocrine secretion of the pancreas and are used by surgeons as prophylactic agents to prevent POPF. Two RCTs observed a reduced incidence of POPF in patients undergoing PD [47,48]. Nonetheless, there have also been reports showing no reduced occurrence of POPF after PD using somatostatin analogs [49], and some controversy also exists about their cost-effectiveness. However, they may consequently be cost-effective for prevention in high-risk patients.

Total pancreatectomy (TP) may also decrease perioperative morbidity and mortality by eradicating the risk of POPF. A retrospective cohort from Capretti et al. showed that patients with FRS ≥ 7 undergoing TP had commendatory short-term outcomes as an alternative to performing a high-risk pancreatic anastomosis [50]. Recent studies confirm the enhanced perioperative outcomes and postoperative quality of life after TP, presumptively due to centralization at high-volume centers, improvements in long-acting insulin, the improved development of pancreatic enzyme substitution, and the availability of autologous islet cell transplantation [51,52,53,54]. In contrast, patients undergoing TP show worse diabetes-related conditions and a high incidence of gastric complications. As a result of ligation of the right gastric and gastroepiploic veins performing TP, association with splenectomy would also impair the left venous drainage, leaving just the coronary vein and the esophageal plexus as the major routes of gastric venous outflow. It is also important to note that all scores included in this study show high sensitivity but low specificity, which means there will be many false-positive patients for POPF. This is important to consider when the scores are applied in clinical practice, as, e.g., TP is a rather aggressive preventive measure for harmful outcomes with POPF. Hence, it might be a good option in patients that develop a grade C POPF and for which a salvage pancreatectomy would be life-saving.

The type of pancreatic anastomosis might also be an important preventive measure. Pancreaticogastrostomy (PG) and pancreatojejunostomy (PJ) are most popular. A German multicenter RCT reported no significant difference in the incidence of POPF after PJ vs. PG (22% vs. 20%, respectively, *p* = 0.617) [55], while the Verona trial did not only show no differences in terms of POPF, but patients undergoing PG had a significantly higher morbidity risk [56]. Although several types of different types of PJ have been reported, no specific type of anastomosis has been proven to reduce the incidence of POPF when compared to other techniques [57]. As a surgeon’s experience has been described as a risk factor for POPF as well, it is generally recommended to adhere to the best-established surgical technique in one surgeon’s hands to achieve best performance.

Another possible preventive strategy to apply to high-risk patients is the external or internal drainage of the MPD to reduce the leakage rate. A meta-analysis of RCTs showed that the POPF rate was statistically significantly lower in the stented group, with no more complications related to the placement or removal of the duct stent. Additionally, the hospital mortality and hospital stay were lower and shorter in the stented patients [58].

Obviously, one of the limitations of this analysis is its retrospective nature. Secondly, the data in this study are based on a single-center experience, and its results should be verified and confirmed in prospective RCTs.

Performing preoperative risk calculations using FRS without BL to determine the groups at risk of POPF, early drain removal (1–3 days postoperative) in low-risk patients, and both the administration of somatostatin analogs and the possible stenting of the MPD in high-risk patients or adapting the surgical approach or anastomotic technique could reduce the risk of POPF. This approach is one step closer to personalized medicine in pancreatic surgery.

## 5. Conclusions

Preoperative risk assessment should be the gold standard, and using the FRS excluding blood loss seems to be a good alternative for the original FRS. Implementing preoperative risk assessment could reduce the risk of POPF by developing a personalized postoperative protocol according to the patient’s risk using different preventive strategies.

## Figures and Tables

**Table 1:** Overview of the preoperative (gray) and intraoperative scores for postoperative pancreatic fistulas with different variables included in each score.

Author	Perri et al. [7]	Roberts et al. [24]	Callery et al. [20] (FRS)	Mungroop et al. [18] (a-FRS)	Mungroop et al. [19] (ua-FRS)	FRS without BL
Country	Italy	England	USA	Multiple ^b^	Pan-European ^c^	
Published	2021	2014	2013	2019	2021	
N	1022 ^a^	325 ^a^	445 ^a^	2850 ^a^	952 ^a^	
AUC	0.675	0.832	0.942	0.750	0.750	
Stratification	Low (8%), intermediate (21%), high (32%)	Low (<5%), intermediate (5–29%), high (30–55%)	Negligible (0%), low (0–5%), intermediate (5–20%), high (>20%)	Low (0–5%), intermediate (5_20%), high (>20%)	Low (0–5%), intermediate (5–20%), high (>20%)	Negligible (0%), low (0–5%), intermediate (5–20%), high (>20%)
ASA	●					
Gender					●	
BMI	●	●		●	●	
Histopathology			●			●
MPD intraoperative			●			
MPD radiological	●	●		●	●	●
BL			●			
Pancreatic texture intraoperative			●			
Pancreatic texture radiological				●	●	●

^a^ includes validation cohort; ^b^ Netherlands, Italy, USA, UK; ^c^ countries not specified; a-FRS, adapted fistula risk score; ASA, American Society of Anesthesiologists; AUC, area under the curve; BL, blood loss; BMI, body mass index; FRS, fistula risk score; MPD, mean pancreatic duct; POPF, postoperative pancreatic fistula; ua-FRS, updated alternative fistula risk score.

**Table 2:** Overview of study population.

Study Group n = 196			
Variable	Condition	Median/n	Range/%
Age at time of surgery (years)		69	26–90
Gender			
	Male	97	49%
	Female	99	51%
BMI		24.6	19.0–31.0
ASA			
	1	10	5%
	2	92	47%
	3	94	48%
Blood loss (mL)		350	40–3000
Surgery duration (min)		539	440–638
Pancreatic anastomosis			
	PJ	195	99%
Pancreatic texture radiological			
	Soft	70	36%
	Hard	126	64%
Malignant lesion		154	79%
	PDAC	91	46%
	Ampullary carcinoma	52	27%
	Cholangiocarcinoma	11	6%
Premalignant		25	13%
	IPMN	9	5%
	MCN	10	5%
	NET	6	3%
Pancreatitis		17	8%
Histological preservation method			
	Surgery	80	41%
	EUS	93	48%
	ERCP	18	9%
	CT guided	5	2%
ICU stay (days)		1	0–55
MPD diameter radiological (mm)		4	1–18
30-day postoperative mortality		3	2%
Clavien–Dindo Classification			
	Grade 0	61	31%
	Grade I	32	16%
	Grade II	74	38%
	Grade IIIa	7	4%
	Grade IIIb	12	6%
	Grade IVa	6	3%
	Grade IVb	1	1%
	Grade V	3	2%
POPF			
	Grade A	4	2%
	Grade B	39	20%
	Grade C	7	4%
PPH			
	Yes	20	10%
	No	176	90%
DGE			
	Yes	42	21%
	No	154	79%
Bile leak			
	Yes	6	3%
	No	190	97%
Intraabdominal abscess			
	Yes	19	10%
	No	177	90%

ASA, American Society of Anesthesiologists; BMI, body mass index; DGE, delayed gastric emptying; ERCP, endoscopic retrograde cholangiopancreatography; EUS, endoscopic ultrasound; ICU, intensive care unit; IPMN, intraductal papillary mucinous neoplasm; MCN, mucinous cystic neoplasm; MPD, mean pancreatic duct; NET, neuroendocrine tumor; PDAC, pancreatic ductal adenocarcinoma; PJ, pancreatojejunostomy; POPF, postoperative pancreatic fistula; PPH, post pancreatectomy hemorrhage.

**Table 3:** Univariate and multivariate logistic regression analyses of preoperative predictors for POPF among the cohort.

		Univariate			Multivariate		
Variables	Condition	OR	95% CI	*p*-Value	OR	95% CI	*p*-Value
Callery et al. [20] *(FRS)*							
Histological diagnosis							
	PDAC/pancreatitis						
	Others	3.53	1.77–7.07	<0.001			
Pancreatic texture							
	Soft	6.86	3.31–14.23	<0.001	5.00	1.95–12.80	<0.001
	Hard						
Blood loss (mL)							
	≤400						
	401–700	1.72	0.79–3.74	0.175			
	701–1000	1.52	0.37–6.19	0.561			
	>1000	1.52	0.37–6.19	0.561			
MPD (mm)							
	≥5						
	4	2.41	0.62–9.35	0.202			
	3	10.23	3.72–28.11	<0.001	7.54	2.33–24.41	<0.001
	2	6.59	2.27–19.16	<0.001			
	≤1	25.33	2.00–321.31	0.013			
Mungroop et al. [18] *(a-FRS)*							
BMI		1.00	0.98–1.02	0.938			
MPD (mm)		0.63	0.50–0.79	<0.001	0.73	0.58–0.91	0.006
Pancreatic texture							
	Soft	6.86	3.31–14.23	<0.001	4.37	2.01–9.50	<0.001
	Hard						
Mungroop et al. [19] *(ua-FRS)*							
Gender							
	Male	2.06	1.04–4.06	0.037	2.35	1.09–5.07	0.029
	Female						
BMI		1.00	0.98–1.02	0.938			
MPD (mm)		0.50	0.37–0.68	<0.001	0.60	0.43–0.83	0.002
Pancreatic texture							
	Soft	6.86	3.31–14.23	<0.001	4.39	1.99–9.72	<0.001
	Hard						
Perri et al. [7]							
BMI							
	<25						
	≥25	1.52	0.78–2.96	0.217			
ASA				0.387			
	1						
	2	0.87	0.21–3.63	0.849			
	3	0.55	0.13–2.35	0.422			
MPD (mm)							
	<5	6.94	2.78–17.35	<0.001	6.83	2.72–17.16	<0.001
	≥5						
Roberts et al. [24]							
BMI		1.00	0.98–1.02	0.938			
MPD (mm)		0.63	0.50–0.79	<0.001	1.00	0.97–1.02	<0.001
*FRS without BL*							
Histological diagnosis							
	PDAC/pancreatitis						
	Others	3.53	1.77–7.07	<0.001			
Pancreatic texture							
	Soft	6.86	3.31–14.23	<0.001	3.83	1.62–9.06	0.002
	Hard						
MPD (mm)							
	≥5	2.41	0.62–9.35	0.202			
	4	10.23	3.72–28.11	<0.001	6.47	2.22–18.88	<0.001
	3	6.59	2.27–19.16	<0.001			
	2	25.33	2.00–321.31	0.013	19.62	1.29–297.36	0.032
	≤1						

a-FRS, adapted fistula risk score; ASA, American Society of Anesthesiologists; BL, blood loss; BMI, body mass index; CI; confidence interval; FRS, fistula risk score; MPD, mean pancreatic duct; OR, odds ratio; PDAC, pancreatic ductal adenocarcinoma; POPF, postoperative pancreatic fistula; ua-FRS, updated alternative fistula risk score.

**Table 4:** POPF rate according to different scoring systems.

	AUC ^a^	AUC ^b^	AUC ^c^	POPF (%)			*p*	95% CI	Sensitivity	Specificity	PPV	NPV
				Low	Medium	High						
Callery et al. [20] (FRS)	0.862	0.862	0.844	4.5	31.5	89.4	<0.001	0.89–0.97	95.2	61.5	43.6	97.6
Mungroop et al. [18] (a-FRS)	0.757	0.738	0.745	5.2	31.2	61.9	<0.001	0.72–0.86	91.3	50.3	36.8	94.8
Mungroop et al. [19] (ua-FRS)	0.733	0.689	0.792	0	10.7	43.8	<0.001	0.74–0.87	100	5.5	25.1	100
Perri et al. [7]	0.693	0.633	0.719	7.4	34.4	37.2	<0.001	0.62–0.78	87	51	35.7	92.6
Roberts et al. [24]	0.614	0.591	0.667	2.6	28.6	37.5	<0.001	0.66–0.81	95.7	34	29	97.4
FRS without blood loss	0.783	0.732	0.885	4.9	28.6	52.4	<0.001	0.72–0.86	91.3	52.4	37.5	95.1

^a^ all patients; ^b^ malignant lesions; ^c^ premalignant lesions; a-FRS, adapted fistula risk score; FRS, fistula risk score; POPF, postoperative pancreatic fistula; ua-FRS, updated alternative fistula risk score.

## Data Availability

The data that support the findings of this study are available from the corresponding author, upon reasonable request.
